# (4′-Acet­yloxy-1,3,1′-trioxo-1,3,4,4a,4b,5,6,7,9,9a-deca­hydro­spiro­[indene-2,9′-pyrano[4,3-*a*]pyrrolizin]-3′-yl)methyl acetate

**DOI:** 10.1107/S1600536813029826

**Published:** 2013-11-13

**Authors:** N. Latha, J. Naga Siva Rao, R. Raghunathan, G. Divya, S. Lakshmi

**Affiliations:** a Research Department of Physics, SDNB Vaishnav College for Women, Chennai 600 044, India; b Department of Organic Chemistry, University of Madras, Chennai 600 025, India

## Abstract

In the title compound, C_23_H_23_NO_8_, the dihedral angle between the five- and six-membered rings of the indene-dione moiety is 3.09 (13)°. The mean plane of the five-membered ring (which has a flat envelope conformation with the spiro C atom as the flap) is inclined to the mean plane of the central five-membered ring of the pyrrolizine unit by 76.48 (12)°. This central ring has a twist conformation on the N—C(spiro) bond. The outer ring of the pyrrolizine unit has an envelope conformation with the N atom as the flap. The mean planes of these two fused rings are inclined to one another by 65.28 (15)°. The pyran ring has a screw-boat conformation and its mean plane makes a dihedral angle of 29.50 (11)° with the mean plane of the central five-membered ring of the pyrrolizine unit. In the crystal, mol­ecules are linked *via* C—H⋯O hydrogen bonds, forming two-dimensional networks lying parallel to the *ab* plane.

## Related literature

For related structures, see: Gayathri *et al.* (2005 ▶); Govind *et al.* (2004 ▶); Kalyanasundaram *et al.* (2005 ▶); Kumar *et al.* (2006 ▶); Satis Kumar *et al.* (2007 ▶) Selvanayagam *et al.* (2005 ▶); Seshadri *et al.* (2003 ▶).
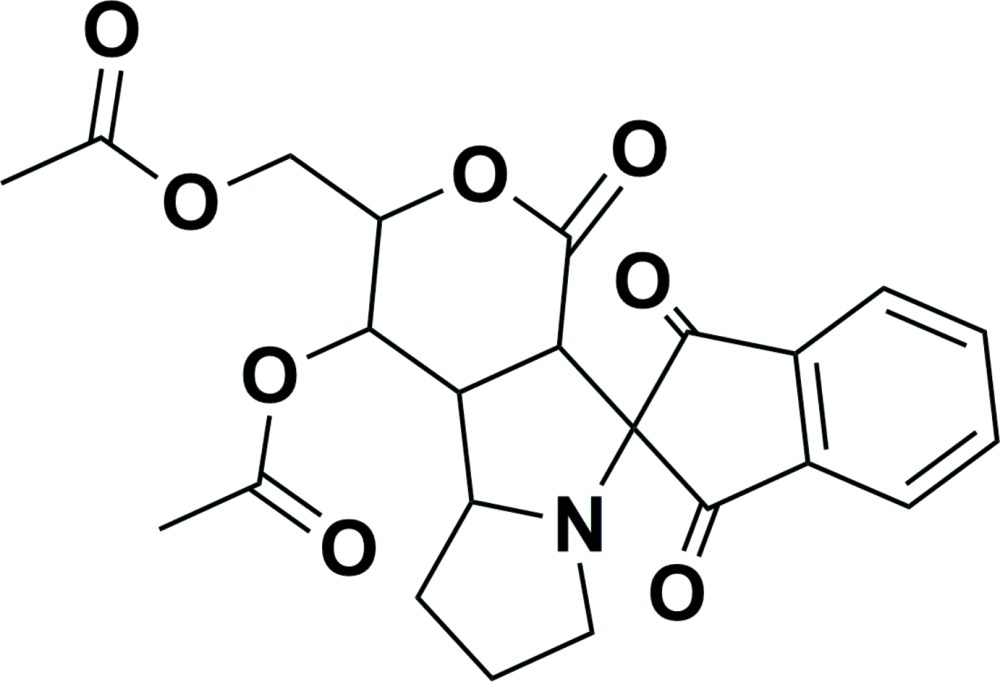


## Experimental

### 

#### Crystal data

C_23_H_23_NO_8_
*M* *_r_* = 441.42
Orthorhombic, 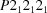

*a* = 10.4817 (4) Å
*b* = 13.4904 (5) Å
*c* = 15.1639 (5) Å
*V* = 2144.21 (13) Å^3^
*Z* = 4
Mo *K*α radiation
μ = 0.10 mm^−1^
*T* = 295 K
0.35 × 0.30 × 0.25 mm

#### Data collection

Bruker Kappa APEXII CCD diffractometer
Absorption correction: multi-scan (*SADABS*; Bruker, 2004 ▶) *T*_min_ = 0.964, *T*_max_ = 0.974
15087 measured reflections
5845 independent reflections
4256 reflections with *I* > 2σ(*I*)
*R*_int_ = 0.032

#### Refinement

*R*[*F* ^2^ > 2σ(*F* ^2^)] = 0.052
*wR*(*F* ^2^) = 0.146
*S* = 1.03
5845 reflections
292 parameters
H-atom parameters constrained
Δρ_max_ = 0.46 e Å^−3^
Δρ_min_ = −0.21 e Å^−3^

### 

Data collection: *APEX2* (Bruker, 2004 ▶); cell refinement: *APEX2* and *SAINT* (Bruker, 2004 ▶); data reduction: *SAINT* and *XPREP* (Bruker, 2004 ▶); program(s) used to solve structure: *SIR92* (Altomare *et al.*, 1993 ▶); program(s) used to refine structure: *SHELXL97* (Sheldrick, 2008 ▶); molecular graphics: *ORTEP-3 for Windows* (Farrugia, 2012 ▶); software used to prepare material for publication: *PLATON* (Spek, 2009 ▶).

## Supplementary Material

Crystal structure: contains datablock(s) I, New_Global_Publ_Block. DOI: 10.1107/S1600536813029826/zs2277sup1.cif

Structure factors: contains datablock(s) I. DOI: 10.1107/S1600536813029826/zs2277Isup2.hkl

Additional supplementary materials:  crystallographic information; 3D view; checkCIF report

## Figures and Tables

**Table 1:** Hydrogen-bond geometry (Å, °)

*D*—H⋯*A*	*D*—H	H⋯*A*	*D*⋯*A*	*D*—H⋯*A*
C3—H3⋯O6^i^	0.93	2.59	3.401 (4)	147
C14—H14⋯O2^ii^	0.98	2.33	3.310 (3)	178

Symmetry codes: (i) 
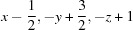
; (ii) 
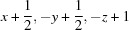
.

**Fig. 1.**
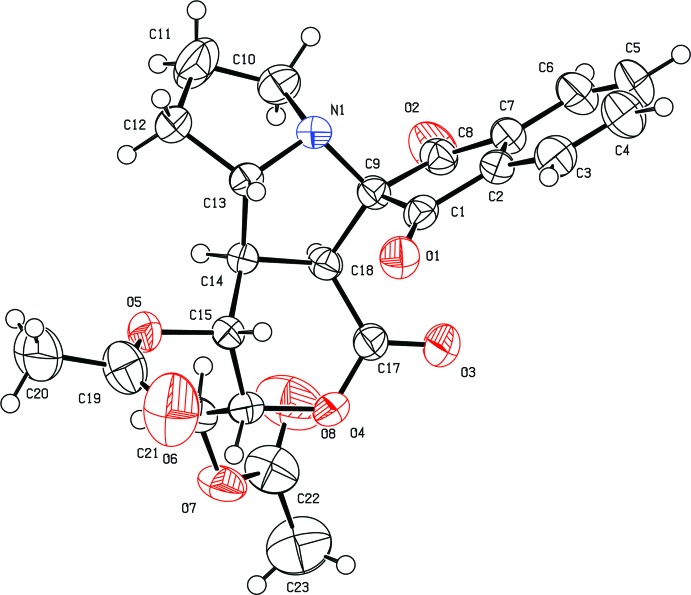
The molecular structure of the title compound with the numbering scheme. Displacement ellipsoids are drawn at the 50% probability level.

**Fig. 2.**
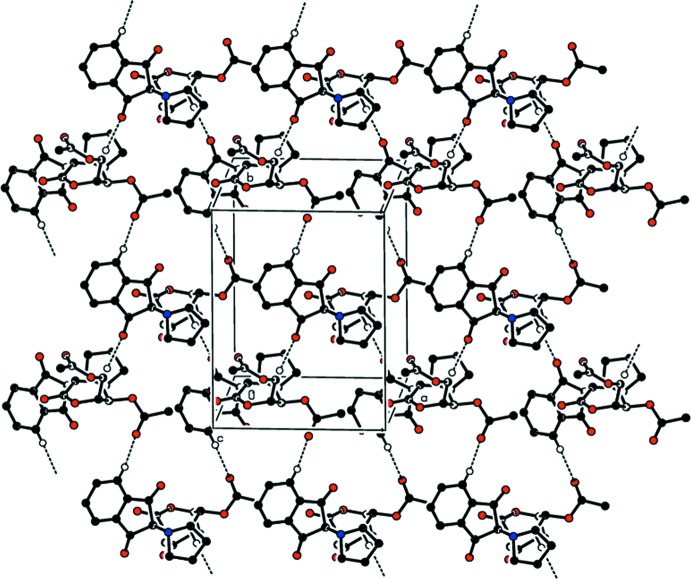
The packing of the title compound, viewed down the c axis.

## supplementary crystallographic information

### 1. Comment

The pyrrolidine skeleton occurs in many families of biologically important compounds. The resulting functionality due to ease of substitution and modification at several positions has been utilized to synthesize compounds with antimicrobial and antifungal properties (Selvanayagam *et al.*, 2005). Derivatives of pyrrolidine are very useful in preventing and treating rheumatoid arthritis, asthma and allergies. They also possess anticonvulsant, anti-influenza (Gayathri *et al.*, 2005) and antivirus activities (Kumar *et al.*, 2006). The spiro (indole-pyrrolidine) ring system is a frequently encountered structural motif in many pharmacologically important alkaloids (Seshadri *et al.*, 2003). In view of its biological activities, the structure determination of the title compound, C_23_H_23_NO_8_ was completed by X-ray diffraction. In this compound the sum of the angles at N1 of the pyrrolidine moiety (325.7°) is consistent with *sp*^3^ hybridization (Kalyanasundaram *et al.*, 2005). The dihedral angle between the five and six-membered rings of the indanone moiety is 3.5 (1)° (Satis Kumar *et al.*, 2007). In the benzene ring of the indole system, the endocyclic angles at C3 and C6 are contracted to 117.5 (3) and 117.1 (3)°, respectively, while those at C4, C5 and C7 are expanded to 122.1 (3), 121.4 (4) and 121.6 (2)°, respectively. This may be due to the fusion of the indole and benzene ring systems where the strain results in angular distortion (Govind *et al.*, 2004). The dihedral angle between the mean planes of the indole system and the pyrrolidine moiety is 77.3 (1)°. The spiro junction at C9 in the indanone group deviates from the mean plane of the C1 – C8 ring by 0.2465 Å. Weak C—H···O intermolecular hydrogen bonds (Table 1) generate a one-dimensional chain structure extending along the *a* axis (Fig. 2).

### 2. Experimental

To a solution of ninhydrin (1 equiv) and proline (1.4 equiv) in dry toluene, α,β-unsaturated sugar lactone was added under a nitrogen atmosphere. The solution was refluxed for 15 h under Dean-Stark reaction conditions to give a cycloadduct. After completion of the reaction indicated by TLC, the solvent was evaporated under reduced pressure. The residual mass was extracted with dichloromethane and water. The organic layer was dried with anhydrous sodium sulfate and concentrated *in vacuo*. The crude mass was purified by column chromatography using hexane/EtOAc (8:2) as an eluent. Crystals suitable for X-ray diffraction were obtained from ethyl acetate solution using a slow evaporation method.

### 3. Refinement

The positions of all the hydrogen atoms were identified from the difference electron density map and were allowed to ride on the parent atoms in calculated positions with distances C—H = 0.93 – 0.98 Å and *U*_iso_(H) = 1.2*U*_eq_(C) for non–methyl groups and *U*_iso_(H) = 1.5*U*_eq_(C) for the methyl groups. The absolute structure was not determined since no strong anamalous scattering atoms are present. However, the configuration for the six trivially named chiral centres [C9(*R*),C13(*S*),C14(*R*),C15(*R*), C16(*R*),C18(*S*)] was consistent with the Flack absolute parameter [0.1 (11) for 2550 Friedel pairs] (Flack, 1983).

### Crystal data

**Table:**

C_23_H_23_NO_8_	*D*_x_ = 1.367 Mg m^−^^3^
*M**_r_* = 441.42	Melting point: 463.15 K
Orthorhombic, *P*2_1_2_1_2_1_	Mo *K*α radiation, λ = 0.71073 Å
Hall symbol: P 2ac 2ab	Cell parameters from 3596 reflections
*a* = 10.4817 (4) Å	θ = 2.7–24.2°
*b* = 13.4904 (5) Å	µ = 0.10 mm^−^^1^
*c* = 15.1639 (5) Å	*T* = 295 K
*V* = 2144.21 (13) Å^3^	Block, yellow
*Z* = 4	0.35 × 0.30 × 0.25 mm
*F*(000) = 928	

### Data collection

**Table:**

Bruker Kappa APEXII CCD diffractometer	5845 independent reflections
Radiation source: fine-focus sealed tube	4256 reflections with *I* > 2σ(*I*)
Graphite monochromator	*R*_int_ = 0.032
ω and φ scan	θ_max_ = 29.3°, θ_min_ = 2.4°
Absorption correction: multi-scan (*SADABS*; Bruker, 2004)	*h* = −13→14
*T*_min_ = 0.964, *T*_max_ = 0.974	*k* = −18→17
15087 measured reflections	*l* = −20→20

### Refinement

**Table:**

Refinement on *F*^2^	Secondary atom site location: difference Fourier map
Least-squares matrix: full	Hydrogen site location: inferred from neighbouring sites
*R*[*F*^2^ > 2σ(*F*^2^)] = 0.052	H-atom parameters constrained
*wR*(*F*^2^) = 0.146	*w* = 1/[σ^2^(*F*_o_^2^) + (0.0808*P*)^2^ + 0.0734*P*] where *P* = (*F*_o_^2^ + 2*F*_c_^2^)/3
*S* = 1.03	(Δ/σ)_max_ < 0.001
5845 reflections	Δρ_max_ = 0.46 e Å^−^^3^
292 parameters	Δρ_min_ = −0.21 e Å^−^^3^
0 restraints	Extinction correction: *SHELXL97* (Sheldrick, 2008), Fc^*^=kFc[1+0.001xFc^2^λ^3^/sin(2θ)]^-1/4^
Primary atom site location: structure-invariant direct methods	Extinction coefficient: 0.0075 (16)

### Special details

**Table:**

Geometry. All e.s.d.'s (except the e.s.d. in the dihedral angle between two l.s. planes) are estimated using the full covariance matrix. The cell e.s.d.'s are taken into account individually in the estimation of e.s.d.'s in distances, angles and torsion angles; correlations between e.s.d.'s in cell parameters are only used when they are defined by crystal symmetry. An approximate (isotropic) treatment of cell e.s.d.'s is used for estimating e.s.d.'s involving l.s. planes.
Refinement. Refinement of *F*^2^ against ALL reflections. The weighted *R*-factor *wR* and goodness of fit *S* are based on *F*^2^, conventional *R*-factors *R* are based on *F*, with *F* set to zero for negative *F*^2^. The threshold expression of *F*^2^ > σ(*F*^2^) is used only for calculating *R*-factors(gt) *etc*. and is not relevant to the choice of reflections for refinement. *R*-factors based on *F*^2^ are statistically about twice as large as those based on *F*, and *R*-factors based on ALL data will be even larger.

### Fractional atomic coordinates and isotropic or equivalent isotropic displacement parameters (Å^2^)

**Table:**

	*x*	*y*	*z*	*U*_iso_*/*U*_eq_	
C1	0.5649 (2)	0.55253 (15)	0.53169 (13)	0.0353 (4)	
C2	0.4474 (2)	0.57154 (16)	0.58231 (13)	0.0397 (5)	
C3	0.3942 (3)	0.6609 (2)	0.60898 (17)	0.0557 (6)	
H3	0.4353	0.7208	0.5984	0.067*	
C4	0.2786 (3)	0.6575 (3)	0.6515 (2)	0.0727 (9)	
H4	0.2409	0.7165	0.6696	0.087*	
C5	0.2171 (3)	0.5700 (3)	0.6681 (2)	0.0754 (9)	
H5	0.1396	0.5709	0.6979	0.090*	
C6	0.2679 (3)	0.4801 (2)	0.64149 (19)	0.0610 (7)	
H6	0.2256	0.4207	0.6520	0.073*	
C7	0.3843 (2)	0.48261 (18)	0.59866 (15)	0.0422 (5)	
C8	0.4589 (2)	0.39934 (16)	0.56199 (15)	0.0399 (5)	
C9	0.5885 (2)	0.43985 (15)	0.53381 (12)	0.0333 (4)	
C10	0.6960 (3)	0.32253 (18)	0.63652 (17)	0.0514 (6)	
H10A	0.6746	0.2743	0.5915	0.062*	
H10B	0.6399	0.3132	0.6867	0.062*	
C11	0.8337 (3)	0.3136 (3)	0.6633 (3)	0.0814 (10)	
H11A	0.8429	0.3268	0.7259	0.098*	
H11B	0.8647	0.2472	0.6514	0.098*	
C12	0.9074 (2)	0.3875 (2)	0.61118 (16)	0.0523 (6)	
H12A	0.9684	0.3545	0.5731	0.063*	
H12B	0.9530	0.4324	0.6499	0.063*	
C13	0.80871 (19)	0.44379 (16)	0.55663 (13)	0.0354 (4)	
H13	0.8279	0.5149	0.5570	0.042*	
C14	0.7914 (2)	0.40667 (14)	0.46069 (12)	0.0323 (4)	
H14	0.8301	0.3408	0.4551	0.039*	
C15	0.84684 (19)	0.47376 (15)	0.39158 (13)	0.0347 (4)	
H15	0.8241	0.5427	0.4046	0.042*	
C16	0.7979 (2)	0.44573 (17)	0.30034 (13)	0.0395 (5)	
H16	0.8386	0.4898	0.2574	0.047*	
C17	0.5883 (2)	0.44448 (18)	0.36714 (13)	0.0407 (5)	
C18	0.64580 (19)	0.39741 (15)	0.44802 (12)	0.0327 (4)	
H18	0.6252	0.3266	0.4462	0.039*	
C21	0.8282 (3)	0.34081 (19)	0.27453 (14)	0.0498 (6)	
H21A	0.9199	0.3322	0.2702	0.060*	
H21B	0.7963	0.2955	0.3191	0.060*	
C22	0.6594 (3)	0.2683 (2)	0.1934 (2)	0.0675 (8)	
C23	0.6042 (4)	0.2586 (3)	0.1044 (3)	0.0961 (12)	
H23A	0.5525	0.3156	0.0918	0.144*	
H23B	0.5525	0.2000	0.1017	0.144*	
H23C	0.6716	0.2540	0.0618	0.144*	
N1	0.68615 (17)	0.42488 (12)	0.60171 (11)	0.0359 (4)	
O1	0.62976 (15)	0.61244 (11)	0.49445 (11)	0.0465 (4)	
O2	0.42539 (19)	0.31479 (13)	0.55496 (14)	0.0628 (5)	
O3	0.47660 (17)	0.46279 (18)	0.36294 (13)	0.0657 (6)	
O4	0.66215 (15)	0.46391 (12)	0.29762 (10)	0.0449 (4)	
O5	0.98345 (14)	0.46223 (12)	0.39493 (11)	0.0467 (4)	
O7	0.76969 (19)	0.31937 (14)	0.19122 (10)	0.0555 (5)	
O8	0.6156 (3)	0.2369 (2)	0.26117 (19)	0.1039 (9)	
O6	1.0109 (3)	0.6120 (2)	0.3367 (2)	0.1059 (10)	
C19	1.0550 (3)	0.5394 (2)	0.3717 (2)	0.0647 (7)	
C20	1.1927 (3)	0.5187 (3)	0.3888 (3)	0.0936 (12)	
H20A	1.2266	0.5685	0.4275	0.140*	
H20B	1.2387	0.5197	0.3340	0.140*	
H20C	1.2014	0.4547	0.4157	0.140*	

### Atomic displacement parameters (Å^2^)

**Table:**

	*U*^11^	*U*^22^	*U*^33^	*U*^12^	*U*^13^	*U*^23^
C1	0.0355 (11)	0.0371 (10)	0.0332 (9)	0.0019 (8)	−0.0057 (8)	−0.0016 (7)
C2	0.0389 (12)	0.0496 (12)	0.0307 (9)	0.0089 (9)	−0.0007 (8)	−0.0046 (8)
C3	0.0657 (17)	0.0560 (14)	0.0455 (12)	0.0181 (12)	−0.0007 (12)	−0.0107 (11)
C4	0.080 (2)	0.082 (2)	0.0564 (16)	0.0323 (18)	0.0119 (16)	−0.0186 (15)
C5	0.0557 (18)	0.109 (3)	0.0616 (17)	0.0244 (18)	0.0206 (14)	−0.0050 (17)
C6	0.0493 (15)	0.0819 (19)	0.0516 (14)	0.0019 (13)	0.0140 (12)	0.0005 (13)
C7	0.0353 (12)	0.0551 (13)	0.0362 (10)	0.0026 (9)	0.0031 (9)	−0.0019 (9)
C8	0.0376 (12)	0.0435 (11)	0.0386 (10)	−0.0044 (9)	0.0032 (9)	0.0003 (9)
C9	0.0330 (10)	0.0349 (9)	0.0321 (9)	0.0012 (8)	0.0019 (8)	−0.0017 (7)
C10	0.0583 (15)	0.0448 (12)	0.0509 (12)	0.0055 (11)	0.0016 (11)	0.0127 (10)
C11	0.063 (2)	0.087 (2)	0.094 (2)	0.0138 (17)	−0.0084 (17)	0.0475 (19)
C12	0.0408 (13)	0.0784 (17)	0.0376 (11)	0.0115 (12)	−0.0050 (10)	0.0017 (11)
C13	0.0331 (11)	0.0421 (10)	0.0309 (9)	0.0009 (8)	−0.0022 (8)	0.0002 (8)
C14	0.0319 (10)	0.0361 (9)	0.0290 (9)	0.0054 (8)	0.0015 (7)	0.0022 (7)
C15	0.0305 (10)	0.0401 (10)	0.0335 (9)	0.0010 (8)	0.0030 (8)	0.0047 (8)
C16	0.0364 (12)	0.0508 (12)	0.0312 (9)	0.0014 (9)	−0.0004 (8)	0.0069 (8)
C17	0.0340 (12)	0.0523 (12)	0.0356 (10)	0.0024 (9)	−0.0030 (9)	−0.0058 (8)
C18	0.0318 (10)	0.0364 (9)	0.0301 (9)	−0.0031 (8)	0.0015 (8)	−0.0025 (7)
C21	0.0525 (15)	0.0626 (15)	0.0345 (11)	0.0040 (12)	0.0034 (10)	−0.0056 (9)
C22	0.077 (2)	0.0620 (17)	0.0632 (17)	−0.0153 (15)	0.0023 (16)	−0.0129 (14)
C23	0.107 (3)	0.094 (3)	0.087 (2)	−0.024 (2)	−0.026 (2)	−0.022 (2)
N1	0.0372 (10)	0.0400 (9)	0.0305 (8)	0.0029 (7)	0.0012 (7)	0.0026 (7)
O1	0.0456 (9)	0.0380 (8)	0.0561 (9)	−0.0010 (6)	0.0024 (8)	0.0077 (7)
O2	0.0607 (12)	0.0479 (10)	0.0799 (12)	−0.0178 (9)	0.0183 (10)	−0.0063 (9)
O3	0.0377 (10)	0.1093 (16)	0.0501 (10)	0.0117 (10)	−0.0087 (8)	−0.0007 (10)
O4	0.0383 (9)	0.0628 (10)	0.0337 (7)	0.0047 (7)	−0.0058 (6)	0.0071 (7)
O5	0.0296 (8)	0.0591 (10)	0.0512 (9)	0.0012 (7)	0.0026 (7)	0.0098 (8)
O7	0.0634 (12)	0.0696 (11)	0.0336 (8)	−0.0089 (9)	0.0063 (8)	−0.0084 (7)
O8	0.103 (2)	0.121 (2)	0.0884 (17)	−0.0511 (17)	0.0194 (16)	0.0001 (16)
O6	0.0768 (17)	0.0691 (15)	0.172 (3)	−0.0177 (13)	0.0071 (18)	0.0219 (17)
C19	0.0461 (16)	0.0670 (18)	0.081 (2)	−0.0131 (13)	0.0071 (14)	−0.0054 (15)
C20	0.0402 (17)	0.150 (4)	0.091 (3)	−0.0208 (19)	0.0006 (16)	0.004 (2)

### Geometric parameters (Å, º)

**Table:**

C1—O1	1.198 (3)	C13—H13	0.9800
C1—C2	1.474 (3)	C14—C15	1.502 (3)
C1—C9	1.540 (3)	C14—C18	1.543 (3)
C2—C3	1.388 (3)	C14—H14	0.9800
C2—C7	1.392 (3)	C15—O5	1.441 (3)
C3—C4	1.374 (4)	C15—C16	1.523 (3)
C3—H3	0.9300	C15—H15	0.9800
C4—C5	1.367 (5)	C16—O4	1.445 (3)
C4—H4	0.9300	C16—C21	1.502 (3)
C5—C6	1.384 (5)	C16—H16	0.9800
C5—H5	0.9300	C17—O3	1.198 (3)
C6—C7	1.382 (4)	C17—O4	1.334 (3)
C6—H6	0.9300	C17—C18	1.507 (3)
C7—C8	1.478 (3)	C18—H18	0.9800
C8—O2	1.198 (3)	C21—O7	1.434 (3)
C8—C9	1.525 (3)	C21—H21A	0.9700
C9—N1	1.466 (3)	C21—H21B	0.9700
C9—C18	1.543 (3)	C22—O8	1.202 (4)
C10—N1	1.482 (3)	C22—O7	1.347 (4)
C10—C11	1.504 (4)	C22—C23	1.474 (5)
C10—H10A	0.9700	C23—H23A	0.9600
C10—H10B	0.9700	C23—H23B	0.9600
C11—C12	1.488 (4)	C23—H23C	0.9600
C11—H11A	0.9700	O5—C19	1.331 (3)
C11—H11B	0.9700	O6—C19	1.206 (4)
C12—C13	1.526 (3)	C19—C20	1.493 (5)
C12—H12A	0.9700	C20—H20A	0.9600
C12—H12B	0.9700	C20—H20B	0.9600
C13—N1	1.477 (3)	C20—H20C	0.9600
C13—C14	1.549 (3)		
			
O1—C1—C2	127.1 (2)	C18—C14—C13	105.01 (15)
O1—C1—C9	125.77 (19)	C15—C14—H14	109.0
C2—C1—C9	107.16 (17)	C18—C14—H14	109.0
C3—C2—C7	120.4 (2)	C13—C14—H14	109.0
C3—C2—C1	129.7 (2)	O5—C15—C14	107.16 (16)
C7—C2—C1	109.86 (18)	O5—C15—C16	109.85 (17)
C4—C3—C2	117.5 (3)	C14—C15—C16	110.72 (17)
C4—C3—H3	121.2	O5—C15—H15	109.7
C2—C3—H3	121.2	C14—C15—H15	109.7
C5—C4—C3	122.0 (3)	C16—C15—H15	109.7
C5—C4—H4	119.0	O4—C16—C21	111.1 (2)
C3—C4—H4	119.0	O4—C16—C15	108.38 (17)
C4—C5—C6	121.4 (3)	C21—C16—C15	113.55 (18)
C4—C5—H5	119.3	O4—C16—H16	107.9
C6—C5—H5	119.3	C21—C16—H16	107.9
C7—C6—C5	117.1 (3)	C15—C16—H16	107.9
C7—C6—H6	121.5	O3—C17—O4	119.0 (2)
C5—C6—H6	121.5	O3—C17—C18	121.4 (2)
C6—C7—C2	121.6 (2)	O4—C17—C18	119.58 (18)
C6—C7—C8	128.7 (2)	C17—C18—C14	117.57 (17)
C2—C7—C8	109.67 (19)	C17—C18—C9	111.96 (17)
O2—C8—C7	127.0 (2)	C14—C18—C9	104.48 (15)
O2—C8—C9	125.3 (2)	C17—C18—H18	107.5
C7—C8—C9	107.73 (18)	C14—C18—H18	107.5
N1—C9—C8	112.07 (16)	C9—C18—H18	107.5
N1—C9—C1	105.23 (16)	O7—C21—C16	109.2 (2)
C8—C9—C1	102.51 (16)	O7—C21—H21A	109.8
N1—C9—C18	105.61 (16)	C16—C21—H21A	109.8
C8—C9—C18	116.74 (17)	O7—C21—H21B	109.8
C1—C9—C18	114.27 (16)	C16—C21—H21B	109.8
N1—C10—C11	103.7 (2)	H21A—C21—H21B	108.3
N1—C10—H10A	111.0	O8—C22—O7	122.0 (3)
C11—C10—H10A	111.0	O8—C22—C23	127.0 (3)
N1—C10—H10B	111.0	O7—C22—C23	111.0 (3)
C11—C10—H10B	111.0	C22—C23—H23A	109.5
H10A—C10—H10B	109.0	C22—C23—H23B	109.5
C12—C11—C10	107.5 (2)	H23A—C23—H23B	109.5
C12—C11—H11A	110.2	C22—C23—H23C	109.5
C10—C11—H11A	110.2	H23A—C23—H23C	109.5
C12—C11—H11B	110.2	H23B—C23—H23C	109.5
C10—C11—H11B	110.2	C9—N1—C13	104.98 (15)
H11A—C11—H11B	108.5	C9—N1—C10	115.28 (18)
C11—C12—C13	105.6 (2)	C13—N1—C10	105.39 (16)
C11—C12—H12A	110.6	C17—O4—C16	121.06 (16)
C13—C12—H12A	110.6	C19—O5—C15	117.8 (2)
C11—C12—H12B	110.6	C22—O7—C21	116.6 (2)
C13—C12—H12B	110.6	O6—C19—O5	122.5 (3)
H12A—C12—H12B	108.7	O6—C19—C20	126.7 (3)
N1—C13—C12	104.62 (17)	O5—C19—C20	110.6 (3)
N1—C13—C14	106.06 (16)	C19—C20—H20A	109.5
C12—C13—C14	115.31 (18)	C19—C20—H20B	109.5
N1—C13—H13	110.2	H20A—C20—H20B	109.5
C12—C13—H13	110.2	C19—C20—H20C	109.5
C14—C13—H13	110.2	H20A—C20—H20C	109.5
C15—C14—C18	110.17 (16)	H20B—C20—H20C	109.5
C15—C14—C13	114.52 (16)		
			
O1—C1—C2—C3	−9.8 (4)	O5—C15—C16—O4	−176.53 (16)
C9—C1—C2—C3	171.2 (2)	C14—C15—C16—O4	65.3 (2)
O1—C1—C2—C7	166.7 (2)	O5—C15—C16—C21	59.5 (2)
C9—C1—C2—C7	−12.3 (2)	C14—C15—C16—C21	−58.7 (2)
C7—C2—C3—C4	0.0 (4)	O3—C17—C18—C14	−160.9 (2)
C1—C2—C3—C4	176.2 (2)	O4—C17—C18—C14	20.9 (3)
C2—C3—C4—C5	0.4 (5)	O3—C17—C18—C9	−39.9 (3)
C3—C4—C5—C6	−1.0 (5)	O4—C17—C18—C9	141.9 (2)
C4—C5—C6—C7	1.0 (5)	C15—C14—C18—C17	6.6 (2)
C5—C6—C7—C2	−0.5 (4)	C13—C14—C18—C17	130.41 (18)
C5—C6—C7—C8	−178.8 (3)	C15—C14—C18—C9	−118.18 (17)
C3—C2—C7—C6	0.0 (3)	C13—C14—C18—C9	5.6 (2)
C1—C2—C7—C6	−176.9 (2)	N1—C9—C18—C17	−154.84 (17)
C3—C2—C7—C8	178.6 (2)	C8—C9—C18—C17	79.9 (2)
C1—C2—C7—C8	1.7 (2)	C1—C9—C18—C17	−39.7 (2)
C6—C7—C8—O2	8.2 (4)	N1—C9—C18—C14	−26.6 (2)
C2—C7—C8—O2	−170.3 (2)	C8—C9—C18—C14	−151.83 (17)
C6—C7—C8—C9	−171.9 (2)	C1—C9—C18—C14	88.6 (2)
C2—C7—C8—C9	9.6 (2)	O4—C16—C21—O7	52.9 (2)
O2—C8—C9—N1	−83.9 (3)	C15—C16—C21—O7	175.42 (19)
C7—C8—C9—N1	96.2 (2)	C8—C9—N1—C13	166.03 (17)
O2—C8—C9—C1	163.8 (2)	C1—C9—N1—C13	−83.32 (18)
C7—C8—C9—C1	−16.1 (2)	C18—C9—N1—C13	37.90 (19)
O2—C8—C9—C18	38.1 (3)	C8—C9—N1—C10	50.5 (2)
C7—C8—C9—C18	−141.80 (19)	C1—C9—N1—C10	161.20 (18)
O1—C1—C9—N1	80.7 (2)	C18—C9—N1—C10	−77.6 (2)
C2—C1—C9—N1	−100.27 (18)	C12—C13—N1—C9	−156.46 (17)
O1—C1—C9—C8	−161.9 (2)	C14—C13—N1—C9	−34.13 (19)
C2—C1—C9—C8	17.1 (2)	C12—C13—N1—C10	−34.3 (2)
O1—C1—C9—C18	−34.6 (3)	C14—C13—N1—C10	88.03 (19)
C2—C1—C9—C18	144.34 (17)	C11—C10—N1—C9	151.2 (2)
N1—C10—C11—C12	−23.7 (4)	C11—C10—N1—C13	35.9 (3)
C10—C11—C12—C13	2.9 (4)	O3—C17—O4—C16	178.3 (2)
C11—C12—C13—N1	19.1 (3)	C18—C17—O4—C16	−3.5 (3)
C11—C12—C13—C14	−97.0 (3)	C21—C16—O4—C17	86.9 (2)
N1—C13—C14—C15	137.90 (16)	C15—C16—O4—C17	−38.5 (3)
C12—C13—C14—C15	−106.8 (2)	C14—C15—O5—C19	−150.7 (2)
N1—C13—C14—C18	16.9 (2)	C16—C15—O5—C19	89.0 (3)
C12—C13—C14—C18	132.2 (2)	O8—C22—O7—C21	−4.3 (5)
C18—C14—C15—O5	−167.74 (16)	C23—C22—O7—C21	175.2 (3)
C13—C14—C15—O5	74.2 (2)	C16—C21—O7—C22	−100.0 (3)
C18—C14—C15—C16	−47.9 (2)	C15—O5—C19—O6	−10.8 (5)
C13—C14—C15—C16	−166.04 (17)	C15—O5—C19—C20	173.5 (3)

### Hydrogen-bond geometry (Å, º)

**Table:**

*D*—H···*A*	*D*—H	H···*A*	*D*···*A*	*D*—H···*A*
C3—H3···O6^i^	0.93	2.59	3.401 (4)	147
C14—H14···O2^ii^	0.98	2.33	3.310 (3)	178

Symmetry codes: (i) *x*−1/2, −*y*+3/2, −*z*+1; (ii) *x*+1/2, −*y*+1/2, −*z*+1.
